# Bridging Breeds: Transcriptomic Insights into Immune Traits of Yili, Thoroughbred, and Kazakh Horses

**DOI:** 10.3390/life15101496

**Published:** 2025-09-23

**Authors:** Tongliang Wang, Xixi Yang, Chuankun Wang, Jianwen Wang, Jun Meng, Xinkui Yao, Yaqi Zeng, Wanlu Ren

**Affiliations:** 1 College of Animal Science, Xinjiang Agricultural University, Urumqi 830052, China; wtl13639911402@163.com (T.W.); xxyang2022@126.com (X.Y.); wck94214@163.com (C.W.); wjw1262022@126.com (J.W.); mengjun@xjau.edu.cn (J.M.); yaoxinkui@xjau.edu.cn (X.Y.); 2 Xinjiang Key Laboratory of Horse Breeding and Exercise Physiology, Xinjiang Agricultural University, Urumqi 830052, China; 3 Horse Industry Research Institute, Xinjiang Agricultural University, Urumqi 830052, China

**Keywords:** blood parameters, Yili horse, immune regulation, RNA sequencing, horse breeding strategies

## Abstract

Background: Studying the genetic characteristics and molecular mechanisms of immune regulation in horses is of great significance for protecting their genetic resources, improving breeding strategies, and enhancing their disease resistance, thereby ensuring their healthy performance in both sports and production. Aims/objectives: This study investigates the genetic characteristics and molecular mechanisms underlying immune regulation in Yili horses, comparing them with Thoroughbreds and Kazakh horses. Methods: Blood samples from each breed were analyzed for physiological, biochemical, and immune indices alongside transcriptome sequencing to identify differentially expressed genes (DEGs). Results: The results revealed significant differences in neutrophil counts, monocytes, red blood cell parameters, glucose levels, and immunoglobulins (IgA, IgG, IgM) among breeds. Yili horses exhibited intermediate values for most parameters, aligning more closely with Thoroughbreds. Transcriptomic analysis identified 3574 DEGs, enriched in immune-related pathways such as platelet activation, antigen processing, and cytokine signaling. Key genes, including TNFRSF14, IFIT3, and IL21R, correlated with immune traits, highlighting hybrid vigor in Yili horses. Functional enrichment underscored pathways like IL-17 signaling and NF-κB regulation, linking genetic differences to immune adaptability. Conclusions: These findings provide molecular insights into breed-specific immune traits, supporting strategies to enhance disease resilience in Yili horses while preserving their athletic performance. This study underscores the importance of integrating transcriptomic and phenotypic data for informed breeding practices in equine conservation and improvement.

## 1. Introduction

Horses are among the most widely distributed terrestrial mammals on Earth [1], showcasing remarkable genetic adaptability to diverse environments and external conditions [2,3]. The existing horse breeds are the result of natural adaptation but were also shaped over time by human selection and societal demands into relatively stable forms. These eventually became domesticated animals through taming and training [1]. Demands for diverse equine traits in modern society led to the development of numerous breeds [4,5], categorized based on breeding levels into local breeds, cultivated breeds, and developed breeds.

Thoroughbreds originated in the UK after centuries of selective breeding and are renowned for their exceptional athletic performance [6]. They have a trim but muscular body structure with strong cardiac output suitable for short bursts of speed. Thoroughbreds introduced to China (Zhaosu) have adapted well to the higher altitude (2000 m) and temperate continental climate. The Kazakh horse, an ancient local breed unique to Xinjiang, China, and Kazakhstan, is characterized by strong disease resistance, adaptability, and tolerance to rough feed [7]. The Yili horse, a superior breed developed in China, combines the disease resistance and adaptability traits of Kazakh mares with the athletic performance of Thoroughbred and Orlov trotter sires. The Yili breed holds domestic records in speed racing, trotting, and endurance events [8]. In immune function research, blood is the target tissue as it carries the various immune cells throughout the body and is a crucial part of the immune defense system [9]. Physiological and biochemical blood parameters are key indicators of an animal’s health and are commonly used to assess overall condition and detect disease. The immune response, which is closely related to health status, can be evaluated by measuring serum immunoglobulin and cytokine concentrations [10], and antibody levels reflect humoral immune capacity [11]. Studies have found that WBC counts exhibit slight differences among different horse breeds. For example, hot-blooded horses, including Arabian and Thoroughbred horses, tend to have slightly higher WBC counts than cold-blooded horses, such as draft horses and ponies [12].

A study on the landrace wild hybrid pig, Min pig, and Largewhite pig revealed that IL-4 levels were highest in Min pigs, TNF-α levels were highest in wild hybrid pigs, and both landrace wild hybrid and Min pigs showed significantly higher IgA, IgM, and IgG levels than Largewhite pigs, indicating stronger immunity in the former breeds [13]. Similarly, studies on the immune differences between Mongolian horses and Thoroughbreds showed that Mongolian horses had significantly higher levels of globulin, IgA, and IgG in their blood. Comparative genetic analyses of these breeds also revealed significant differences in genotype distributions [14]. Blood transcriptome analysis is an effective molecular approach for identifying potential biomarkers and biological pathways related to immune system function in both in vivo and in vitro studies of healthy animals with differing immune traits (ITs) [15]. Previous research identified several key immune-related genes in horses. For example, the class II antigens of the major histocompatibility complex (MHC) are highly polymorphic cell surface proteins that play a critical role in triggering immune responses [16], and toll-like receptors (TLRs) on host immune cells are activated by recognition of pathogen-associated molecular patterns (PAMPs) [17].

In recent years, with the accelerated breeding process, the athletic performance of Yili horses has significantly improved, but potentially at the risk of declining resilience. In the interests of conservation biology, this calls for further investigation into the differences in immune traits and responses among horse breeds; we employed transcriptome analysis of blood, a minimally invasive method, for these comparisons. We compared immune indicators and blood transcriptomes of Thoroughbred, Yili, and Kazakh horses to identify immune-related differentially expressed genes (DEGs) and potential pathways, aiming to preserve the resilience of Yili horses during the breeding process and establish a molecular foundation for breeding strategies.

## 2. Materials and Methods

### 2.1. Animals Used in Study, Study Locations, Whole Blood Collection and Storage

All animal experiments were conducted in strict compliance with the ethical principles and guidelines of the Animal Welfare and Ethics Committee of Xinjiang Agricultural University, ensuring adherence to animal welfare standards (Approval No. 2023037, Date: 16 July 2023). The study subjects were selected from Kazakh (n = 3), Thoroughbred (n = 3), and Yili (n = 3) mares in the Yili region of Xinjiang, all 6 years old (the sampling was conducted in June, during the local summer season). The horses had not received any medications or intravenous treatments within the three months prior to the experiment and were in good health. They received about 8 kg of roughage (hay) per day, approximately 4 kg of concentrate, and free access to drinking water. More details are available in Table 1.

### 2.2. Measurement of Blood Physiological and Biochemical Parameters

Five mL blood samples were drawn using vacuum blood collection tubes (Shenyang Baokang Bioengineering Co., Ltd., Shenyang, China) without anticoagulants for measurements of physiological indices. A fully automated, five-classification animal blood cell analyzer (BC-5300 Vet, Mindray, Shenzhen, China) was used to measure the following parameters: white blood cells (WBCs), neutrophils (Neu), lymphocytes (Lym), monocytes (Mon), eosinophils (Eos), basophils (Bas), red blood cells (RBCs), hemoglobin concentration (HGB), hematocrit (HCT), mean corpuscular volume (MCV), mean corpuscular hemoglobin (MCH), mean corpuscular hemoglobin concentration (MCHC), red blood cell distribution width coefficient of variation (RDW-CV), red blood cell distribution width standard deviation (RDW-SD), and platelets (PLTs).

Five milliliter blood samples were collected from the jugular vein of fasting animals using EDTA-K anticoagulant tubes (Shenyang Baokang Bioengineering Co., Ltd., Shenyang, China) centrifuged at 3000 r/min for 10 min at 4 °C to extract plasma for biochemical and immunological index determination. In addition, a fully automated animal biochemical analyzer (BS-240 VET, Mindray, China) was used to measure glucose (GLU), alanine aminotransferase (ALT), aspartate aminotransferase (AST), alkaline phosphatase (ALP), total protein (TP), total cholesterol (TC), urea (UREA), and albumin (ALB).

### 2.3. Blood Immune Index Measurement

Five milliliters of fasting blood was drawn from the jugular vein into EDTA-K anticoagulant tubes (Guangzhou Improve Medical, Guangzhou, China), immediately placed on ice, and centrifuged at 3000 r/min (4 °C, 10 min) to obtain plasma. Aliquots were stored at −80 °C within 1 h and shipped on dry ice to Beijing Northern Biotechnology Research Institute Co., Ltd. (Beijing, China) for immune profiling.

IgA, IgG, and IgM were determined in triplicate by endpoint turbidimetry (Kehua Bio-Engineering, Shenzhen, China). Plasma samples (10 μL) were mixed with 250 μL of antibody reagent, incubated 10 min at 37 °C, and the absorbance was read at 340 nm on a Hitachi 7180 auto-analyzer (Hitachi,·Tokyo, Japan). Calibration was performed using the manufacturer’s six-point standard curve.

IL-4, IFN-γ, and TNF-α were quantified in triplicate by sandwich ELISA (Invitrogen, USA) using pre-coated 96-well plates. Aliquots of 100 μL of plasma diluted 1:2 in assay buffer were added to the wells and the plate was incubated for 2 h at RT, followed by sequential 1 h incubations with biotinylated detector antibody, streptavidin-HRP, and TMB substrate. The reaction was stopped with 2 N H_2_SO_4_ and absorbance was read at 450 nm (reference 630 nm) on a BioTek ELx800 plate reader (BioTek, Winooski, VT,·USA). Concentrations were calculated from four-parameter logistic standard curves with the following ranges: IL-4, 2–200 pg/mL; IFN-γ, 4–400 pg/mL; TNF-α, 8–800 pg/mL. The intra-assay CV was <8% and the inter-assay CV was <10%.

### 2.4. Statistical Analysis

Statistical analyses were performed using SPSS 26.0 software (IBM, Armonk, NY, USA). One-way analysis of variance (ANOVA) was conducted, followed by Duncan’s multiple comparison test for intergroup comparisons. Results are presented as mean ± standard deviation (SD), with *p* < 0.05 considered statistically significant.

### 2.5. RNA Extraction, Library Construction, and Quality Control

One 5 mL sample was mixed with TRIzol reagent (Invitrogen, Carlsbad, CA, USA) at a 1:3 ratio and dispensed into 5 mL EDTA-K anticoagulant tubes, then stored at −80 °C for subsequent transcriptomic analysis. RNA library construction and transcriptome sequencing of the nine blood samples were performed by Novogene Co., Ltd. (Beijing, China). RNA was extracted and its integrity assessed using an Agilent 2100 Bioanalyzer. AMPure XP beads were used for screening and PCR amplification to construct the library using the NEBNext Ultra RNA Library Prep kit on an Illumina HiSeq™ 2500 platform. Library quantification and insert size were evaluated with a Qubit 2.0 fluorometer (Thermo Fisher Scientific, Waltham, MA, USA) and Agilent 2100 bioanalyzer (Agilent Technologies Inc., Santa Clara, CA, USA), respectively, with accurate library concentration determined by real-time quantitative PCR (RT-qPCR), showing an effective concentration > 2 nM. Libraries that passed quality control were sequenced using an Illumina HiSeq™ 2500 platform (Illumina Technologies Inc., San Diego, CA, USA) based on the desired data yield.

### 2.6. Sequencing Data Quality Control and Alignment

Image data from CASAVA were converted into sequence data (reads). Sequencing error rates reflected machine accuracy. Low-quality reads and sequences with adapters were filtered out to ensure data reliability. Clean reads were aligned to the *Equus caballus* reference genome (NCBI RefSeq, https://ftp.ncbi.nlm.nih.gov/genomes/refseq/vertebrate_mammalian/Equus_caballus/latest_assembly_versions/GCF_002863925.1_EquCab3.0/GCF_002863925.1_EquCab3.0_genomic.fna.gz, accessed on 13 May 2025) using HISAT2 software (Center for Computational Biology, Johns Hopkins University) for genomic location information.

### 2.7. Differential Gene Analysis

Statistical analysis was conducted on the gene expression data from the nine samples. The raw reads of non-normalized genes were used as experimental data. After differential expression analysis, the Benjamini–Hochberg method was applied to adjust the *p*-values for multiple hypothesis testing and to obtain the false discovery rate (FDR). The criteria for identifying DEGs were |log2Fold Change| ≥ 1.5 and FDR < 0.05.

### 2.8. Clustering and Enrichment Analysis of Differential Genes

Fragments per kilobase of transcript per million mapped reads (FPKM) values of genes were clustered to group genes with similar expression patterns. DEGs from pairwise comparisons of the three breeds were subjected to GO (gene ontology) and KEGG (Kyoto Encyclopedia of Genes and Genomes) enrichment analysis using Cluster Profiler software. Pearson correlation coefficients were used to analyze the relationship between DEGs and immune indices, with *p* < 0.05 indicating significant correlations.

### 2.9. Real-Time Quantitative PCR (qRT-PCR) Validation

GAPDH (glyceraldehyde-3-phosphate dehydrogenase) has been widely used as an internal reference gene in fluorescence quantification (especially qPCR) for a long time, mainly due to some stable expression characteristics it was believed to have in the past [18].

To validate DEGs, six immune-related genes were randomly selected for qRT-PCR (Table 2). Gene primers were designed using Primer-BLAST based on cDNA sequences downloaded from the NCBI database, with GAPDH as the reference gene. Gene expression was calculated using the 2^−ΔΔCt^ method to assess the accuracy and reproducibility of the transcriptome sequencing data.

### 2.10. Correlation Analysis

Pearson correlation analysis was performed between the gene expression levels (FPKM values) of differentially expressed genes obtained from transcriptome sequencing and blood physiological, biochemical, and immune parameters. Correlations with |cor| > 0.3 and *p*-value < 0.05 were considered significant. The correlation results were then imported into Cytoscape 3.9.1 (Cytoscape Consortium, San Diego, CA, USA) to construct a network diagram.

## 3. Results

### 3.1. Blood Physiological and Biochemical Parameters

As shown in Table 3, the Neu value in Kazakh horses was significantly lower than that in Thoroughbreds and Yili horses (*p* < 0.05). In contrast, the Mon value in Kazakh horses was significantly higher than in the other two breeds. The RBC count in Thoroughbreds was significantly higher than in Kazakh horses (*p* < 0.05) but showed no significant difference from Yili horses (*p* > 0.05). HGB, HCT, MCHC, and PLT were significantly higher in Thoroughbreds and Yili horses (*p* < 0.05) and lowest in Kazakh horses. No significant differences (*p* > 0.05) were observed among the three breeds for the remaining blood parameters. As shown in Table 4, GLU levels differed significantly among the three horse breeds. Specifically, Thoroughbreds had the highest GLU content compared to Kazakh and Yili horses (*p* < 0.05). The ALT, AST, ALP, TP, TC, and UREA levels in Yili horses were between those of Kazakh and Thoroughbred horses, but no significant differences were found among the three breeds (*p* > 0.05). Additionally, ALB levels were the lowest in Yili horses among the three breeds (*p* > 0.05).

### 3.2. Immune Indicators

As shown in Table 5, the levels of IFN-γ and IL-4 in Thoroughbreds were significantly higher than those in Kazakh horses (*p* < 0.05), while no significant differences were observed between Yili horses and the other two breeds (*p* > 0.05). The level of TNF-α in Yili horses was significantly higher than that in Kazakh horses (*p* < 0.05), with no significant differences between Thoroughbreds and the other two breeds (*p* > 0.05). The IgA, IgG, and IgM levels in Kazakh horses were significantly lower than those in Yili horses and Thoroughbreds (*p* < 0.05), while no significant differences were observed between Yili horses and Thoroughbreds (*p* > 0.05).

### 3.3. RNA Extraction and Quality Control

The raw reads from the nine samples exceeded 4.2 × 10^7^ for each, and after filtering out low-quality reads, the clean bases were all above 5.34 G, with an error rate of 0.03%. The sequencing data quality showed that Q20 values were approximately 97%, and Q30 values ranged between 93.41% and 94.18%, indicating high-quality sequencing data. Quality control results are shown in Table S1.

### 3.4. Mapping Reads to the Reference Genome

The clean reads from the nine samples were aligned to the *Equus caballus* reference genome using HISAT2. Most clean reads were successfully mapped, with alignment rates ranging from 78.52% to 95.32%. Of these, 74.94% to 91.74% were uniquely mapped. The high alignment rates indicate that the selected reference genome was suitable for further analysis (Table S2).

### 3.5. Analysis of DEGs Among Thoroughbred, Yili, and Kazakh Horses

**Figure 1:**
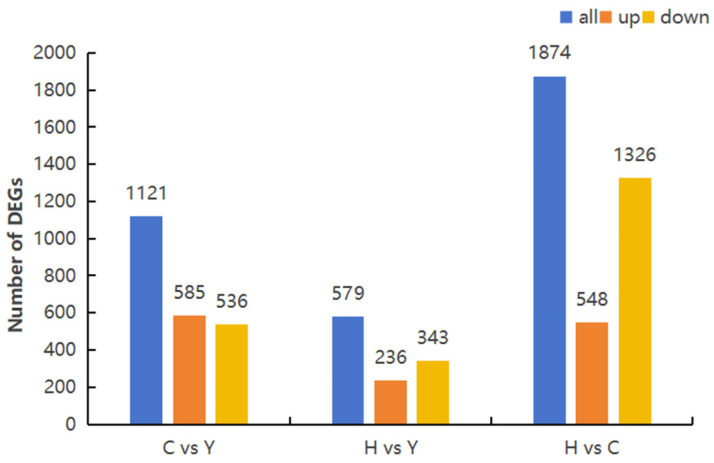
Comparison of DEG numbers among Thoroughbred, Yili, and Kazakh horses.

A total of 3574 DEGs were identified using DESeq. Between Thoroughbreds and Yili horses, 1121 DEGs were detected, including 585 upregulated and 536 downregulated genes. Between Kazakh and Yili horses, 579 DEGs were identified, with 236 upregulated and 343 downregulated. The relationships among DEGs across the groups are depicted in Figure 1.

### 3.6. Gene Ontology (GO) Enrichment Analysis of Differentially Expressed Genes

**Figure 2:**
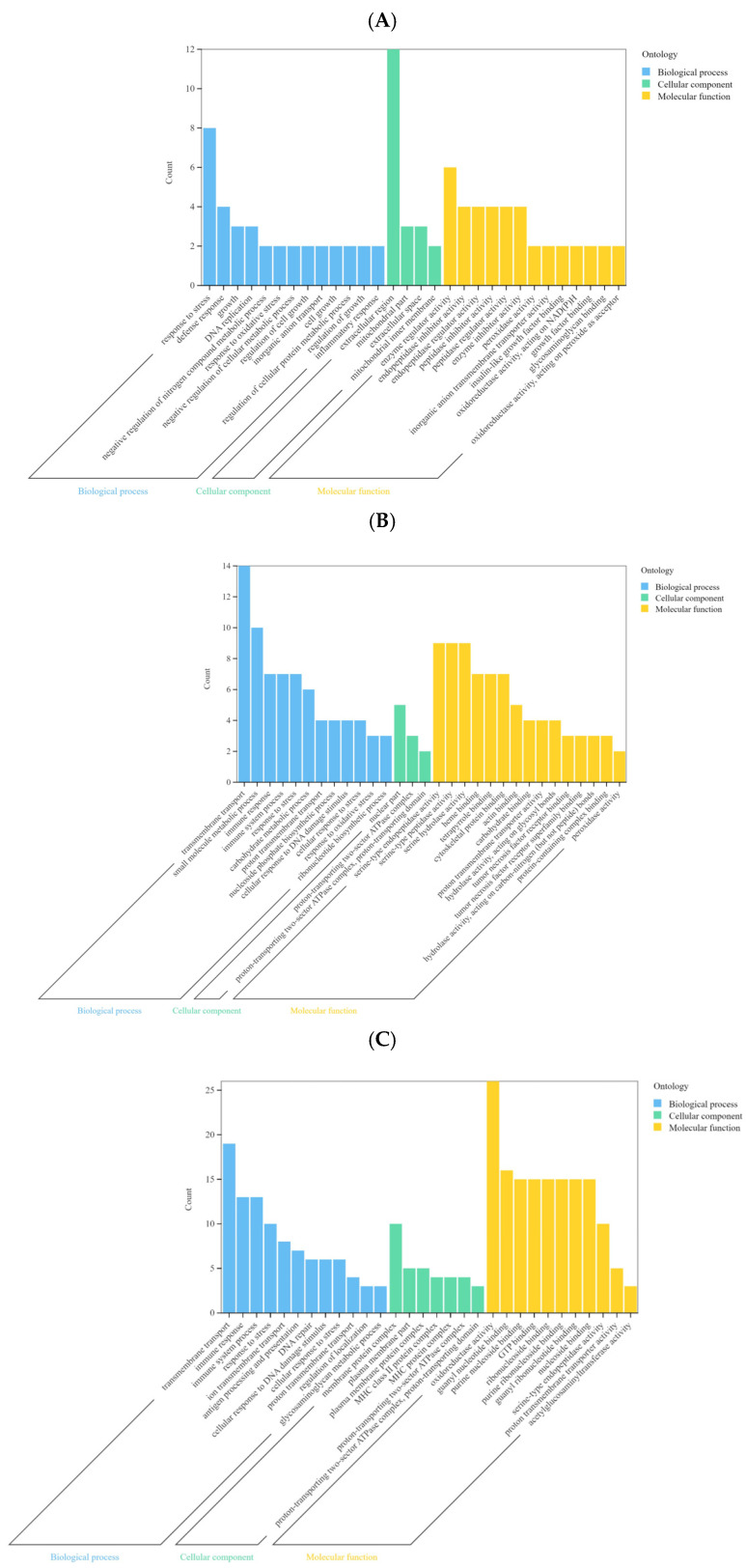
Gene ontology (GO) enrichment analysis of DEGs in different group comparisons. (**A**) Thoroughbreds vs. Yili horses; (**B**) Kazakh vs. Yili horses; (**C**) Kazakh vs. Thoroughbred horses.

To investigate the functional roles of DEGs in the gene expression profiles of the three horse breeds, GO enrichment analysis was performed for pairwise comparisons: Thoroughbreds vs. Yili horses, Kazakh vs. Yili horses, and Kazakh vs. Thoroughbred horses (Figure 2A–C). DEGs in Thoroughbreds vs. Yili horses were enriched in terms such as response to stress, response to oxidative stress, defense response, and inflammatory response. DEGs in Kazakh vs. Yili horses were enriched in transmembrane transport, immune system processes, and serine-type peptidase activity. DEGs in Kazakh vs. Thoroughbreds were enriched in transmembrane transport, immune response, and response to stress.

### 3.7. KEGG Enrichment Analysis of Differentially Expressed Genes

**Figure 3:**
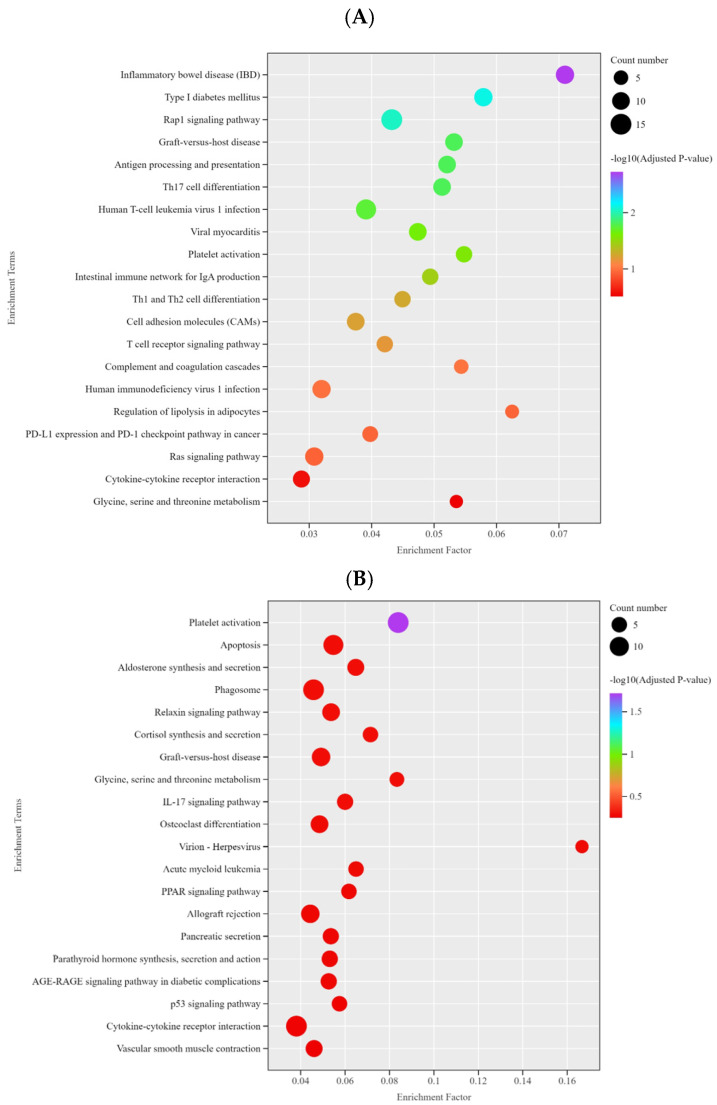

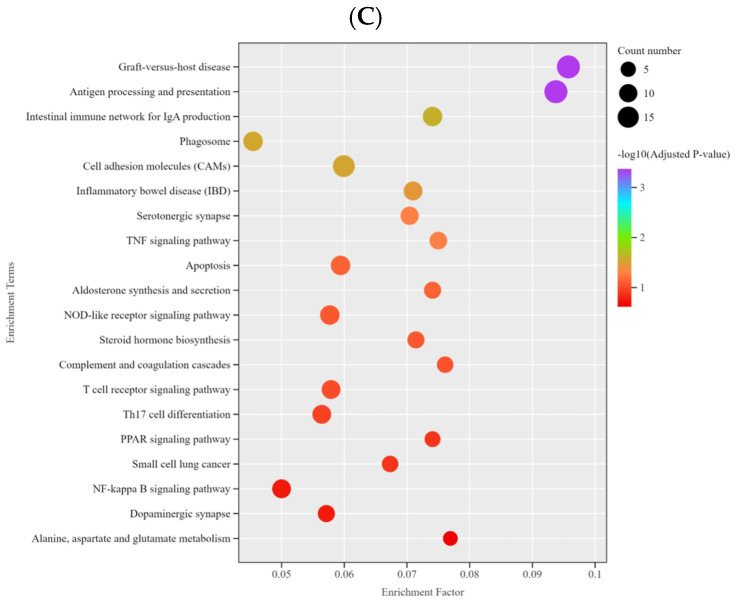
KEGG enrichment analysis bubble plots. (**A**) Thoroughbred vs. Yili horses; (**B**) Kazakh vs. Yili horses; (**C**) Kazakh vs. Thoroughbred horses.

KEGG pathway analysis revealed the top 20 enriched pathways (Figure 3A–C). DEGs between Thoroughbreds and Yili horses were enriched in pathways such as inflammatory bowel disease, type I diabetes mellitus, and Rap1 signaling. DEGs between Kazakh and Yili horses were enriched in platelet activation, apoptosis, and aldosterone synthesis and secretion. DEGs between Kazakh horses and Thoroughbreds were enriched in graft-versus-host disease, antigen processing and presentation, and intestinal immune network for IgA production.

We further categorized and summarized all pathways enriched by differentially expressed genes (DEGs) and found that in the Thoroughbred vs. Yili horse group, the highest number of DEGs was enriched in the immune system pathway, followed by signal transduction and infectious disease: viral pathways, each containing 16 DEGs. In the Kazakh vs. Yili horse group, the endocrine system pathway had the highest number of enriched DEGs (21), followed by the immune system and cell growth and death pathways. In the Kazakh vs. Thoroughbred group, 42 DEGs were enriched in the immune system pathway, indicating that this pathway is particularly important across all three comparison groups.

**Figure 4:**
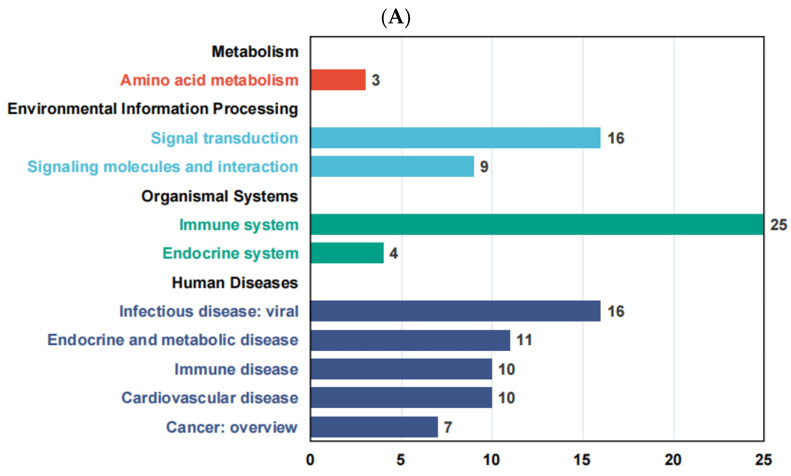

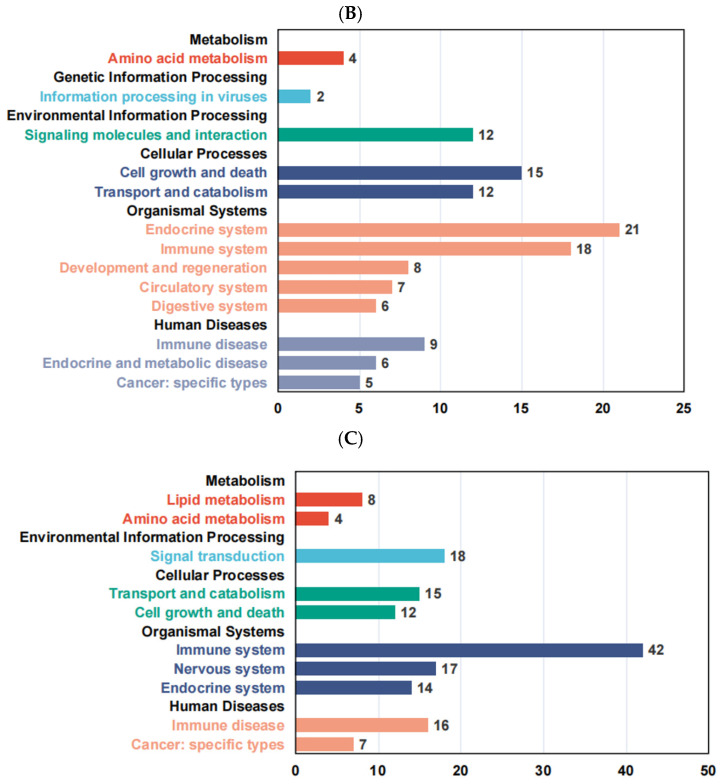
KEGG pathway enrichment result classification summary diagram. (**A**) Thoroughbred vs. Yili horses; (**B**) Kazakh vs. Yili horses; (**C**) Kazakh vs. Thoroughbred horses.

KEGG enrichment analysis identified 19 pathways from the Thoroughbred vs. Yili horses, 16 for Kazakh vs. Yili horses, and 16 for Kazakh vs. Thoroughbred horses (Figure 4A). All pathways were closely associated with immune regulation. In the KEGG enrichment analysis of Thoroughbred vs. Yili horses, pathways involving *LOC111768588* and *IL1β* were found to be related to type I diabetes mellitus and Th17 cell differentiation, respectively. *ITGA2B*, *F2RL3*, *PTGS1*, and *ADCY5* were associated with the platelet activation pathway, while *IL18* was linked to inflammatory bowel disease (IBD) and the cytokine–cytokine receptor interaction pathways (Figure 4A). *ADCY5* and *PTGIR* were also involved in the platelet activation pathway. *TNF*, *IL13*, and *FOS* were associated with the IL-17 signaling pathway, while *TNFRSF14* was linked to pathways like virion–herpesvirus and cytokine–cytokine receptor interaction. For Kazakh vs. Thoroughbred horses (Figure 4B), *MHCB3* was associated with pathways such as phagosome, graft-versus-host disease, and allograft rejection. In addition, *MHCB3*, *DQA*, and *DQB* were linked to antigen processing and presentation, while *DQA*, *FOS*, and *DQB* were involved in Th17 cell differentiation. Notably, *FOS* was associated with multiple pathways, including the TNF signaling pathway, apoptosis, T cell receptor signaling pathway, Th17 cell differentiation, and dopaminergic synapse (Figure 4C).

### 3.8. Protein–Protein Interaction (PPI) Analysis

**Figure 5:**
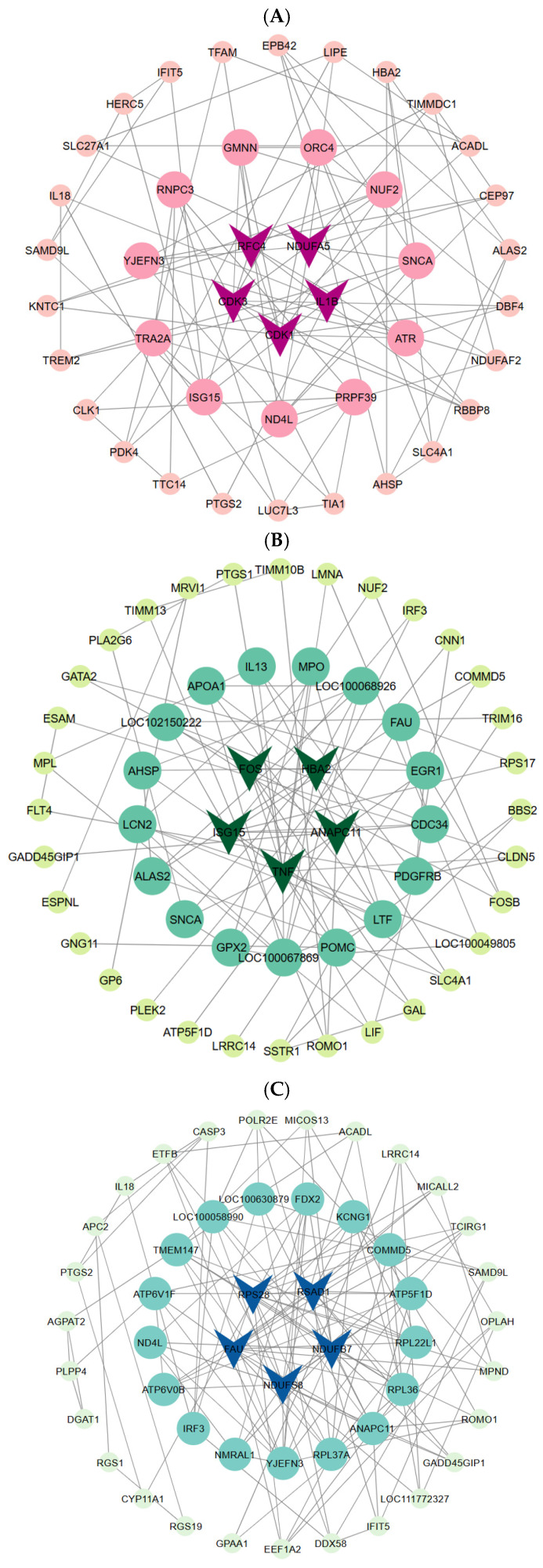
PPI network analysis. (**A**) Thoroughbred vs. Yili horses; (**B**) Kazakh vs. Yili horses; (**C**) Kazakh vs. Thoroughbred horses.

The DEG protein interaction network was constructed based on the STRING database, with isolated nodes having fewer than two connections filtered out to enhance network reliability. In the Thoroughbred vs. Yili horses comparison, RFC4, NDUFA5, CDK3, CDK1, and IL1B exhibited the highest node degrees (Figure 5A). In the Kazakh vs. Yili horses comparison, FOS, HBA2, ANAPC11, TNF, and ISG15 were identified as key nodes (Figure 5B). In the Kazakh vs. Thoroughbred horses comparison, RPS28, RSAD1, NDUFB7, NDUFS8, and FAU were the central nodes (Figure 5C).

### 3.9. RT-qPCR Validation

**Figure 6:**
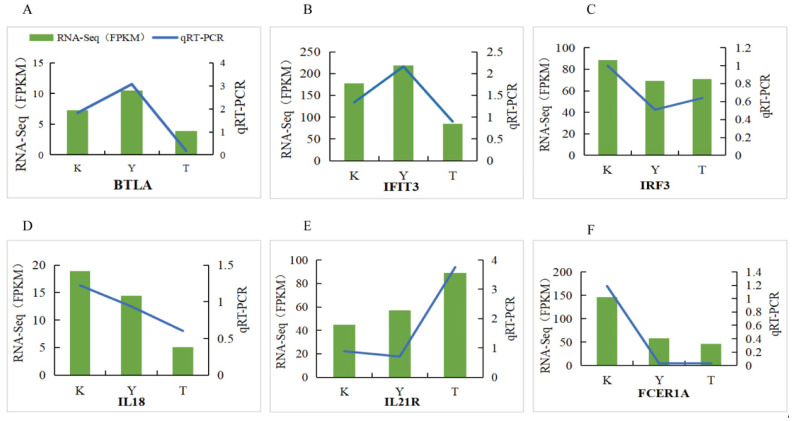
qRT-PCR results for selected DEGs. (**A**) BTLA, (**B**) IFIT3, (**C**) IRF3, (**D**) IL18, (**E**) IL21R, (**F**) FCER1A.

To establish the accuracy of the sequencing data, six mRNAs were randomly selected and analyzed by qRT-PCR. As shown by qRT-PCR in Figure 6, the expression levels and change trends of the six validation genes in the blood of horses of different breeds were the same as those in the results of RNA-seq analysis, indicating that the RNA-seq sequencing data of this experiment were accurate and could be used for further research and analysis.

### 3.10. Correlation Analysis

**Figure 7:**
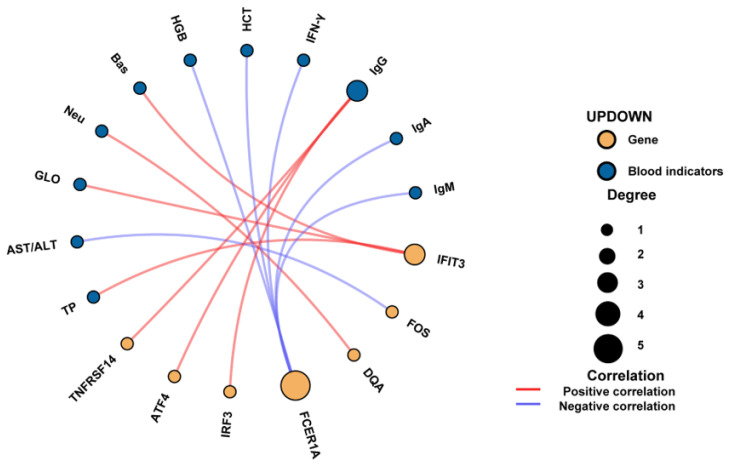
Correlation network diagram between blood parameters and differentially expressed genes.

Correlation analysis (all breeds) was performed between the DEGs from significantly enriched KEGG pathways and the measured blood parameters (including physiological, biochemical, and immune indicators). The results showed that IgG was positively correlated with the genes TNFRSF14, ATF4, and IRF3. FCER1A was negatively correlated with HGB, HCT, IFN-γ, IgA, and IgM. IFIT3 showed a positive correlation with GLO, TP, and Bas levels (Figure 7).

## 4. Discussion

The breeding of horses holds significant importance in the history of animal domestication, profoundly transforming human social structures, culture, and lifestyle while fostering a harmonious relationship between humans and horses [19]. The earliest evidence of horse domestication dates back to 3500 BCE [20]. Horses have adapted across regions, leading to diverse breeds classified by genetics, morphology, and use. There are about 547 breeds globally (https://horse.xjau.edu.cn/breeds/list, accessed on 24 July 2024). Blood tests and biochemical indicators are key tools for assessing horses’ health and physiology [21].

Changes in parameters indicate the functional status of specific organs, such as the liver. In this study, all measured parameters fell within the normal reference ranges provided by Li [22] and Frank [23], indicating that all horses in the study were in good health. White blood cells (WBCs) play a crucial role in the immune response of animals. WBC counts and their subtypes can indirectly reflect an animal’s susceptibility, the virulence of invading pathogens, and the nature and severity of diseases [24,25]. Neutrophils (Neu) are responsible for phagocytosing and killing bacteria [26]. Monocytes (Mon) are the largest leukocytes in circulation, maturing into macrophages once they migrate into tissues. In the leukocyte profile, apart from Neu and Mon, no significant differences were found among the breeds for other indicators. A previous study reported breed differences in innate immune traits, including neutrophil and monocyte counts, as well as acute-phase proteins, between Meishan and Large White pigs [27]. Similarly, in our study, Neu counts were higher in Thoroughbreds and Yili horses compared to Kazakh horses, while Mon counts were highest in Kazakh horses. This aligns with previous findings, confirming breed differences in Neu and Mon among Kazakh, Thoroughbred, and Yili horses. Red blood cells (RBCs) play a vital role in oxygen and carbon dioxide transport. As a native Japanese breed, Kiso horses have demonstrated strong adaptability due to their long history of local breeding. Studies suggest that when RBC counts increase without a change in volume, the surface area of the red blood cell membrane expands, accelerating gas diffusion and increasing diffusion capacity. This mechanism allows HGB to optimize gas exchange and improve oxygen utilization efficiency [28]. In Thoroughbreds, post-exercise RBC, HGB, and MCH levels were significantly higher than those in Jeju horses, indicating that increased RBCs enhance oxygen transport capacity during exercise [29]. In our study, RBC, HGB, and HCT levels were significantly lower in Kazakh horses compared to Yili and Thoroughbred horses. This discrepancy may be explained by two factors. First, Kazakh horses have been locally bred in Xinjiang for an extended period, whereas Yili horses have been selectively bred relatively recently, and Thoroughbreds are an introduced breed with a short local breeding history. Thus, the latter two breeds could exhibit compensatory adaptations to the local environment. Secondly, Thoroughbreds were bred to be speed-type racing horses, while Yili horses were created as a versatile riding-draft breed, and each has superior athletic ability [30]. Their hemoglobin exhibits higher affinities for oxygen and their RBC, HGB, and HCT levels are increased to supply oxygen more efficiently to muscle tissues during exercise. The coagulation and hemostatic abilities of the body are primarily derived from the function of platelets (PLT) [31]. PLT also plays a critical role in inflammation and pathological processes. In this study, PLT levels were significantly higher in Thoroughbreds compared to Kazakh and Yili horses. As an introduced breed, Thoroughbreds exhibited PLT levels within the normal range but slightly higher than the local Kazakh breed and selectively bred Yili horses. A higher PLT count may help prevent external injuries and enhance resilience in harsh environments. These findings suggest that as an introduced breed, Thoroughbreds have developed a robust physiological foundation for adapting to new environments and management conditions, while the selectively bred Yili horses display intermediate values for several parameters, aligning more closely with Thoroughbreds than Kazakh horses.

Glucose (GLU) in the blood serves as a crucial substrate for protein synthesis and plays an essential role in maintaining normal cellular functions. Insufficient GLU levels can impair immune function [32]. The capacity for the storage and utilization of GLU is a key factor in exercise metabolism, as physical activity can alter glucose levels [33]. During intense exercise, energy demand increases, leading to accelerated glucose breakdown. The high GLU levels observed in Thoroughbreds may be due to their primary use in speed racing, which requires high glucose consumption during exercise [34]. In contrast, the Thoroughbreds in this study were not training horses, leading to GLU being stored rather than consumed.

IgA is a dimeric antibody that operates at mucosal surfaces. In the equine respiratory tract, it prevents pathogen adherence by coating the epithelium and is actively transported through epithelial cells into the lumen (reviewed in [35]). We therefore measured BALF IgA to evaluate the integrity of the first-line mucosal barrier. Thoroughbreds and Yili horses showed the highest BALF IgA concentrations, suggesting that their airway mucosa was better protected against initial viral or bacterial attachment. IgM is a pentameric antibody that dominates the primary immune response. Its large size confines it mainly to the vascular compartment, but it readily diffuses into the airway lumen when epithelial permeability increases during early infection [35]. We used BALF IgM as a marker of acute, sub-clinical respiratory challenge. Elevated IgM was detected in the two athletic breeds, indicating recent or recurrent exposure to airborne pathogens that had been neutralized without clinical disease. IgG is a monomeric antibody that accounts for 70–80% of serum immunoglobulin and is the principal agent for neutralizing blood-borne pathogens [35]. A proportion of serum IgG transudes into the respiratory lumen, so BALF IgG reflects both systemic antibody production and local vascular leakage. The higher BALF IgG levels recorded in Thoroughbreds and Yili horses are consistent with a more vigorous systemic antibody response, a trait that has been linked to superior genetic fitness and athletic capacity [36,37].

Taken together, the parallel rise of all three antibody isotypes in the athletic breeds supports the interpretation that their respiratory immunity is constitutively upregulated, providing a mechanistic link between superior performance and a lower incidence of respiratory disease.

IFN-γ is an immunomodulatory molecule that enhances immune responses during infection and cancer [38]. It coordinates various protective functions by inducing antiviral activity, enhancing antigen processing and presentation, facilitating leukocyte trafficking, boosting the body’s antibacterial functions, and regulating cell proliferation and apoptosis. The lower levels of IFN-γ in Kazakh horses suggest a strong resistance to infections and bacteria.

TNF-α is a key pro-inflammatory cytokine that can induce tumor cell apoptosis and inhibit tumor proliferation and metastasis. It plays a critical role in various inflammatory diseases, including irritable bowel syndrome (IBS) and inflammatory bowel disease (IBD). Studies have shown that elevated TNF-α levels can influence intestinal motility through neural and endocrine pathways, leading to abdominal pain [39]. Based on blood parameter analysis, the concentration of TNF-α in Yili and Thoroughbred horses was significantly higher than in Kazakh horses, which may also suggest a stronger disease resistance in the local breeds. These elevated TNF-α levels in Yili and Thoroughbred horses could indicate a more robust innate inflammatory response, potentially contributing to their enhanced capacity to counteract pathogens and tissue injury. However, persistently high TNF-α may also imply a heightened susceptibility to chronic inflammatory conditions if not properly regulated. Therefore, the increased TNF-α concentration might reflect a dual role, facilitating rapid immune activation but also necessitating tighter control mechanisms to prevent inflammatory pathology. Further studies should explore the regulatory pathways and functional consequences of elevated TNF-α in these horse breeds to better evaluate their overall immune adaptability and health resilience.

As the performance of the Yili horse breed increases, the gap in racing speed compared to superior foreign breeds is gradually narrowing. However, compared to Kazakh horses, Yili horses have a relatively lower ability to withstand harsh environments, which may increase their disease susceptibility. Consequently, Yili horses may be more prone to developing abdominal pain when external environmental conditions change [40].

To ensure the reliability and validity of the differential expression analysis results in this study, we performed a series of rigorous quality controls and validations. First, all samples passed strict quality control standards, including sequencing quality, alignment rate, and inter-sample correlation analysis. PCA analysis demonstrated good cohesion within biological replicates, and the major variation was driven by experimental grouping rather than batch effects. We employed the standard normalization method of DESeq2 and its built-in statistical model for differential analysis, applying stringent thresholds (FDR < 0.05 and |log2FC| > 1) to identify differentially expressed genes and control the false positive rate. In addition, qRT-PCR validation of six randomly selected differentially expressed genes showed high consistency with the sequencing data, further supporting the reliability of our results. Finally, GO/KEGG enrichment analysis revealed that the differentially expressed genes were significantly enriched in relevant pathways, consistent with the expected biological background of the study, thereby strengthening the biological plausibility of the results.

To investigate differences in blood transcriptomes among introduced, local, and bred horse populations, over 5.34 GB of clean reads per sample were obtained from high-throughput sequencing, yielding 3574 DEGs. Comparisons revealed 1121 DEGs between Thoroughbreds and Yili horses, 1874 DEGs between Kazakh horses and Thoroughbreds, and 579 DEGs between Kazakh and Yili horses. Several immune-related genes were identified. For example, the gene *F2RL3*, which encodes a protein associated with cardiovascular function, is highly expressed in Thoroughbreds. This may affect cardiovascular function and increase the risk of heart remodeling [41].

Conversely, IFIT3, an innate immune modulator [42,43], showed higher expression in Yili and Kazakh horses, highlighting the inheritance of innate immune response capabilities from Kazakh horses. IL21R is not only expressed on fibroblasts but also on T cells, B cells, and macrophages in the blood, where its receptor is present on their surfaces. Studies also indicated that macrophages play a key role in muscle repair [44], with IL21R, expressed in fibroblasts and macrophages [45], being significantly upregulated in early tendon injuries [46]. High IL21R expression in Thoroughbreds suggests susceptibility to musculoskeletal and tendon injuries during strenuous activity.

Functional enrichment analysis of DEGs revealed key differences between the breeds. KEGG analysis for Thoroughbreds and Yili horses highlighted pathways related to disease and immune processes, such as the T cell receptor signaling pathway, which is crucial for antigen recognition and adaptive immunity [47], and platelet activation, vital for vascular integrity and immune responses. These pathways are likely to contribute to the immune system differences between Thoroughbreds and Yili horses.

In comparisons between Kazakh horses and Thoroughbreds, pathways like antigen processing and presentation played essential roles in adaptive immunity, forming part of the defense mechanism against pathogens [48]. Two classical immune pathways—the T cell receptor signaling pathway and NF-kappa B signaling pathway—were also enriched, with the latter being linked to tendon diseases [49]. The intestinal immune network for IgA production was consistent with immune indicator results, suggesting that these pathways contributed to immune differences between Thoroughbreds and Kazakh horses. Studies have shown that the bidirectional signaling pathways involving TNFRSF14 and its ligands, such as BTLA and CD160, can activate pro-inflammatory signals (e.g., via NF-κB) as well as transmit inhibitory signals, thereby regulating T cell activation and immune tolerance. Additionally, TNFRSF14 interacts with BTLA to balance immune activation and suppression, which is of great significance in autoimmune diseases and tumor immune evasion [50]. The expression levels of TNFRSF14, BTLA, and CD160 were higher in Yili horses compared to the other two breeds, with TNFRSF14 showing a significant positive correlation with IgG. Moreover, IgG levels in Yili horses were higher than those in Kazakh horses, suggesting that, as a cultivated breed, Yili horses exhibit hybrid vigor and possess a stronger immune response.

In Kazakh vs. Yili horses, DEGs were enriched in immune-related pathways such as platelet activation, IL-17 signaling, and PPAR signaling. The IL-17 pathway coordinates innate and adaptive immune responses to infections [51], while the PPAR pathway regulates immune cell functions like macrophage activation and polarization [52]. PPARα, in particular, plays a role in gut immunity and microbiota homeostasis, maintaining intestinal mucosal immunity by regulating IL-22 and antimicrobial peptide expression [51]. This highlights the observation that immune differences between Kazakh and Yili horses are primarily related to infection resistance and intestinal health.

The enrichment of these functions underscores the intricate control of immune functions by multiple pathways and signaling factors. Further research is needed to explore these mechanisms, particularly for Yili horses’ genetic improvement. However, this study is limited because of the small sample size owing to the limited population of specific breeds and challenges in obtaining tissue samples from equine immune organs. We identified candidate genes and pathways associated with immune differences among the three breeds, providing markers for breeding disease-resistant Yili horses. Future large-scale studies are necessary to clarify the relationship between these genes and breed-specific immune traits. However, this research does offer valuable insights into immune performance differences across horse breeds and molecular-level data supporting Yili horse breeding efforts.

This study still contains certain limitations.

1. Scarcity and access restrictions of specific breeds:

Among the three breeds examined, Kazakh horses in the Zhaosu County conservation herd are either rare or strictly managed under national preservation regulations. Accessing blood samples from these horses involves complex approval and ethical procedures, making it extremely difficult to obtain more qualified individuals in the short term.

2. Strict inclusion criteria and resource limitations:

To ensure consistency in defining a “healthy” state, we applied stringent health standards (e.g., clinical examination within a specific timeframe, no medication history). These criteria significantly limited the pool of eligible horses. Additionally, financial and timeline constraints of the project rendered sample size expansion unfeasible within the original research plan.

3. Context-specific geographic and historical focus:

This study was designed as an initial exploration of baseline immune gene expression within representative populations under defined geographic origins and management conditions. While adding samples from other sources might increase the sample size, it could also introduce new confounding variables and deviate from the study’s original scope.

Despite the small sample size, we observed consistent and biologically plausible gene expression patterns within and between breeds. These trends offer preliminary evidence for potential immune-related transcriptomic differences under defined conditions and provide hypotheses that can be further validated in larger studies.

## 5. Conclusions

Our study compared immune indicators among three horse breeds with a relatively small sample size. Blood parameters revealed significant differences among horse breeds in white blood cell count, red blood cell count, platelet count, and glucose levels, as well as immunoglobulin and cytokine levels. Transcriptomic analysis identified 3574 DEGs shared among the breeds, along with 38 core signaling pathways and 19 core candidate genes closely related to substance transport, cellular metabolism, and immune processes. We identified candidate genes and signaling pathways that, despite the small sample size, showed consistent effects within breeds and influenced immune traits across different horse breeds. These findings contribute to the conservation, utilization, and innovation of Kazakh horse germplasm resources and provide a theoretical basis for further breeding strategies aimed at improving the quality of Yili horses.

## Figures and Tables

**Table 1:** Composition and nutritional levels of the basal diet (on a dry matter basis).

Item	Content (%)
Ingredients	
Corn	17.28
Wheat bran	5.26
Soybean meal	9.26
Dicalcium phosphate	1.15
Salt	0.63
Premix	0.31
Methionine	0.19
Dried hay	65.92
Total	100
Nutrient levels	
Dry matter	95.31
Crude protein	12.69
Crude fat	1.77
Neutral detergent fiber (NDF)	42.23
Acid detergent fiber (ADF)	37.98
Crude ash	8.16
Calcium (Ca)	0.86
Phosphorus (P)	0.41
Digestible energy (MJ/kg)	9.03

Note: 1. The premix provided the following per kg of the concentrate supplement: VA 14 mg, VB1 21.29 mg, VB2 336.5 mg, VB6 1.27 mg, VD 2.3 mg, VE 857 mg, biotin 6 mg, pantothenic acid 4.56 mg, nicotinamide 12.17 mg, Cu (as copper sulfate) 43.24 mg, Fe (as ferrous sulfate) 111.94 mg, Mn (as manganese sulfate) 183.27 mg, Zn (as zinc sulfate) 176.04 mg, I (as potassium iodide) 29.69 mg, Se (as sodium selenite) 42.29 mg, and Co (as cobalt chloride) 4.06 mg. 2. Nutrient levels are measured values.

**Table 2:** Primer sequences, product sizes, and annealing temperatures for qRT-PCR (Quantitative Reverse Transcription PCR) validation of selected genes.

Label	Gene	Primer Sequences	Product Size (bp)	Annealing Temp (°C)
A	*BTLA*	TCAGCACCATTGTCTGAGCC	86	62.46
		AGCACCAACATGAACCTCCT		
B	*IFIT3*	TGGCCATCGCGACATACTG	118	59.9
		AGGGCAAGGAGAACTTTGACA		
C	*IRF3*	AACCGGAAGGAAGTGTTGCG	87	59.71
		CGCTGGGGTCAGAAACTCAT		
D	*IL18*	GAGCAGTGAGGAGTTCCGTT	184	60.32
		CCAGCGGCCATCTTTATTCC		
E	*IL21R*	CGTGACCTTCTCGGGACTTT	104	59.9
		CTCCCCGCATCTTGTACTGC		
F	*FCER1A*	GGATTGGGACGTCTTCAAGGT	125	58.82
		CCTCGCAGTAATAGGTGCCA		
	*GAPDH*	CTAAGTCGTCTCTGCTGAG	113	59.81

Note: *BTLA*: B and T Lymphocyte Attenuator; *IFIT3*: Interferon-Induced Protein with Tetratricopeptide Repeats 3; *IRF3*: Interferon Regulatory Factor 3; *IL18*: Interleukin 18; *IL21R*: Interleukin 21 Receptor; *FCER1A*: Fc Fragment Of IgE Receptor Ia; *GAPDH*: Glyceraldehyde-3-Phosphate Dehydrogenase.

**Table 3:** Comparison of blood indices of Kazakh, Thoroughbred, and Yili horses.

Index	Kazakh Horses	Thoroughbreds	Yili Horses
WBC (10^9^/L)	9.22 ± 0.47	9.69 ± 1.34	10.48 ± 0.68
Neu # (10^9^/L)	4.01 ± 0.32 ^b^	5.80 ± 1.24 ^a^	5.44 ± 0.16 ^a^
Lym # (10^9^/L)	4.23 ± 0.15	3.28 ± 0.39	4.26 ± 1.00
Mon # (10^9^/L)	0.34 ± 0.06 ^a^	0.18 ± 0.01 ^b^	0.19 ± 0.05 ^b^
Eos # (10^9^/L)	0.52 ± 0.09	0.38 ± 0.20	0.58 ± 0.17
Bas # (10^9^/L)	0.08 ± 0.03	0.04 ± 0.02	0.08 ± 0.02
RBC (10^12^/L)	6.35 ± 1.03 ^b^	8.86 ± 0.87 ^a^	8.40 ± 1.22 ^ab^
HGB (g/L)	107.33 ± 15.18 ^b^	152.00 ± 13.00 ^a^	143.33 ± 18.15 ^a^
HCT (%)	30.80 ± 4.71 ^b^	41.73 ± 0.71 ^a^	39.33 ± 3.94 ^a^
MCV (fL)	48.63 ± 2.29	47.17 ± 2.31	47.07 ± 2.30
MCH (pg)	16.97 ± 0.85	17.20 ± 0.78	17.07 ± 0.38
MCHC (g/L)	349.00 ± 4.58 ^b^	364.33 ± 2.31 ^a^	363.33 ± 10.02 ^a^
RDW-CV (%)	22.50 ± 1.37	21.37 ± 1.17	22.20 ± 0.44
RDW-SD (fL)	37.83 ± 2.74	35.57 ± 0.25	36.80 ± 1.40
PLT (10^9^/L)	104.00 ± 5.57 ^b^	227.67 ± 21.35 ^a^	184.67 ± 23.80 ^a^

Note: Different lowercase superscripts in the same row indicate significant differences at *p* < 0.05. WBC: White Blood Cell Count; Neu: Neutrophil Count; Lym: Lymphocyte Count; Mon: Monocyte Count; Eos: Eosinophil Count; Bas: Basophil Count; RBC: Red Blood Cell Count; HGB: Hemoglobin Concentration; HCT: Hematocrit; MCV: Mean Corpuscular Volume; MCH: Mean Corpuscular Hemoglobin; MCHC: Mean Corpuscular Hemoglobin Concentration; RDW-CV: Red Cell Distribution Width Coefficient of Variation; RDW-SD: Red Cell Distribution Width Standard Deviation; PLT: Platelet Count.

**Table 4:** Comparison of blood biochemical indexes of Kazakh, Thoroughbred, and Yili horses.

Index	Kazakh Horses	Thoroughbreds	Yili Horses
Glu (mmol/L)	3.02 ± 0.3 ^b^	3.71 ± 0.05 ^a^	3.01 ± 0.19 ^b^
ALT (U/L)	5.87 ± 1.10	5.13 ± 0.32	5.5 ± 0.46
AST (U/L)	241.87 ± 36.23	255.37 ± 5.27	247.47 ± 32.10
ALP (U/L)	215.93 ± 14.45	251.43 ± 8.56	240.70 ± 32.92
TP (g/L)	57.93 ± 5.33	50.07 ± 2.49	56.30 ± 13.87
TC (mmol/L)	2.33 ± 0.62	1.92 ± 0.28	2.22 ± 0.57
UREA (mmol/L)	8.51 ± 2.03	7.28 ± 1.73	7.40 ± 3.65
ALB (g/L)	22.33 ± 4.52	25.30 ± 2.56	21.80 ± 6.58

Note: Different lowercase superscripts in the same row indicate significant differences at *p* < 0.05. Glu: Glucose; ALT: Alanine Aminotransferase; AST: Aspartate Aminotransferase; ALP: Alkaline Phosphatase; TP: Total Protein; TC: Total Cholesterol; UREA: Urea; ALB: Albumin.

**Table 5:** Comparison of blood immune indices of Kazakh horses, Yili horses, and Thoroughbreds.

Index	Kazakh Horses	Thoroughbreds	Yili Horses
IFN-γ (pg/mL)	57.93 ± 27.62 ^b^	83.79 ± 36.51 ^a^	73.28 ± 19.31 ^ab^
TNF-α (pg/mL)	23.62 ± 13.14 ^b^	37.56 ± 15.12 ^a^	45.5 ± 22.96 ^a^
IL-4 (pg/mL)	20.38 ± 11.87 ^b^	39.73 ± 17.82 ^a^	32.49 ± 19.03 ^ab^
IgA (g/L)	2.13 ± 0.16 ^b^	3.19 ± 0.23 ^a^	3.09 ± 0.13 ^a^
IgG (g/L)	19.67 ± 0.81 ^b^	21.96 ± 0.82 ^a^	21.59 ± 1.18 ^a^
IgM (g/L)	1.27 ± 0.09 ^b^	1.79 ± 0.13 ^a^	1.78 ± 0.11 ^a^

Note: Different lowercase superscripts in the same row indicate significant differences at *p* < 0.05.

## Data Availability

The original contributions presented in this study are included in the article/Supplementary Materials. Further inquiries can be directed to the corresponding authors.

## Supplementary Materials

The following supporting information can be downloaded at: https://www.mdpi.com/article/10.3390/life15101496/s1, Table S1: Quality control results of RNA-seq data; Table S2: Results of mapping samples to the reference genome.
