# Supplementary material for: Bridging Breeds: Transcriptomic Insights into Immune Traits of Yili, Thoroughbred, and Kazakh Horses
Source: Life (Basel). 2025 Sep 23;15(10):1496. doi: 10.3390/life15101496 (PMC12565139; doi:10.3390/life15101496)
Supplement: Supplementary file 1 [file life-15-01496-s001.zip › life-3866172-supplementary.pdf]

# Supplementary Materials

**Table S1.** Quality control results of RNA-seq data.

| Sample | Raw reads | Clean reads | Clean bases | Error (%) | Q20 (%) | Q30 (%) | GC (%) |
|---|---|---|---|---|---|---|---|
| **K1** | **46481748** | **45542274** | **6.83G** | **0.03** | **97.63** | **93.74** | 53.43 |
| K2 | 43885710 | 43057312 | 6.46G | 0.03 | 97.52 | 93.45 | 54.00 |
| K3 | 45948772 | 45272686 | 6.79G | 0.03 | 97.87 | 94.18 | 54.66 |
| C1 | 45525234 | 44848784 | 6.73G | 0.03 | 97.47 | 93.41 | 58.36 |
| C2 | 42822276 | 42114514 | 6.32G | 0.03 | 97.53 | 93.46 | 56.26 |
| C3 | 45357210 | 44355848 | 6.65G | 0.03 | 97.50 | 93.40 | 55.40 |
| T1 | 47532188 | 46510490 | 6.98G | 0.03 | 97.50 | 93.46 | 52.20 |
| T2 | 47556804 | 40845754 | 6.13G | 0.03 | 97.78 | 93.88 | 57.46 |
| T3 | 47013098 | 45492734 | 6.82G | 0.03 | 97.81 | 93.91 | 52.99 |

**Table S2.** Results of mapping samples to the reference genome.

| Sample | Total reads | Total map | Unique map | Multi-map | Read1 map | Read2 map |
|---|---|---|---|---|---|---|
| K1 | 45542274 | 42896818 (94.19%) | 41398525 (90.9%) | 1498293 (3.29%) | 20789722 (45.65%) | 20608803 (45.25%) |
| K2 | 43057312 | 40619122 (94.34%) | 38754541 (90.01%) | 1864581 (4.33%) | 19435583 (45.14%) | 19318958 (44.87%) |
| K3 | 45272686 | 43153888 (95.32%) | 41533692 (91.74%) | 1620196 (3.58%) | 20810334 (45.97%) | 20723358 (45.77%) |
| T1 | 44848784 | 39994005 (89.18%) | 38460892 (85.76%) | 1533113 (3.42%) | 19315442 (43.07%) | 19145450 (42.69%) |
| T2 | 42114514 | 38300499 (90.94%) | 36874177 (87.56%) | 1426322 (3.39%) | 18514795 (43.96%) | 18359382 (43.59%) |
| T3 | 44355848 | 41650899 (93.9%) | 39874282 (89.9%) | 1776617 (4.01%) | 20025560 (45.15%) | 19848722 (44.75%) |
| Y1 | 46510490 | 43827606 (94.23%) | 42369051 (91.1%) | 1458555 (3.14%) | 21291686 (45.78%) | 21077365 (45.32%) |
| Y2 | 40845754 | 35156577 (86.07%) | 34083683 (83.44%) | 1072894 (2.63%) | 17136946 (41.96%) | 16946737 (41.49%) |
| Y3 | 45492734 | 42924012 (94.35%) | 41465474 (91.15%) | 1458538 (3.21%) | 20828052 (45.78%) | 20637422 (45.36%) |
